# Back to Old Books Toward Affordable Research: Homemade Phenol-Based Reagent for Triphasic RNA Purification

**DOI:** 10.1007/s10528-023-10509-3

**Published:** 2023-09-25

**Authors:** Mahmoud M. Abdelfattah, Yasmine Hosny, Nadia A. Elkady, Eslam M. Abbas, Iman G. Farahat, Nagwa H. A. Hassan, Reham Helwa

**Affiliations:** 1 Molecular Cancer Biology Group, Zoology Department, Faculty of Science, Ain Shams University, Cairo, Egypt; 2 Vertebrate Biology Group, Zoology Department, Faculty of Science, Ain Shams University, Cairo, Egypt; 3 Department of Microbiology, Faculty of Science, Ain Shams University, Cairo, Egypt; 4 Department of Chemistry, Faculty of Science, Ain Shams University, Cairo, Egypt; 5 Department of Pathology- National Cancer Institute, Cairo University, Cairo, Egypt; 6 Present Address: Faculty of Advanced Basic Science, King Salman International University, South Sinai, Ras Sudr, Egypt

**Keywords:** Phenol-based reagent, RNA, Acidic phenol, Homemade purification, Eukaryotic cells

## Abstract

Covid-19 crisis did hit many socio-economic aspects in the whole world. In the scientific research, the problem is getting even worse, since most of materials and consumable are allocated to the health sector. Many research laboratories around the world have big delay in receiving their purchases to accomplish their research projects. In the developing countries, the situation is much more difficult, since most of the funding resources are directed to the Covid-19 crisis and there is a notable increase in reagents’ prices. Therefore, the aim of the present study is to make a homemade reagents for RNA purification from eukaryotic cells/tissues. The homemade phenol-based RNA extraction reagents were prepared using saturated phenol pH 4.3 (adjusted by 0.5 M citrate buffer) and guanidine thiocyanate. To validate the phenol-based reagent, RNA was purified from different biological samples (cell line, tissues, and fungi) using homemade phenol-based versus a commercial one. Concentration of RNA samples extracted from the same number of cells were compared to assess the homemade preparation of phenol-based reagent. In conclusion, homemade phenol-based reagent is cost effective and comparable to the commercial one. Using homemade phenol-based, RNA extraction was successfully purified from different biological sources.

## Introduction

RNA is involved in different bioanalytical experiments such qRT-PCR, RNA sequencing, microarray analysis, and RNA viruses diagnosis. Thus, nucleic acid extraction is a daily lab routine and a crucial step in molecular research and diagnostics (Stears et al. 2003; Tan and Yiap 2009; Wang et al. 2009).

TRI reagent or commercially named TRIzol is a monophasic reagent which is composed of phenol and guanidine thiocyanate (Rio et al. 2010). Guanidine thiocyanate is a strong protein denaturant which has big advantage in RNase activity inhibition (Chomczynski and Sacchi 1987; Dooley and Castellino 1972; Rio et al. 2010). In phenol-based RNA purification method, chloroform is added after dissolving the investigated biological samples. Adding chloroform separates the monolayer to three layers: upper aqueous layer which contains RNA, interstitial layer with DNA, and protein in the lower organic layer (Chomczynski 1993; Rio et al. 2010).

During the Covid-19 pandemic, many labs including our lab experienced delayed orders and price surge of many consumables. As subsequent results, we have many behindered research projects due to this shortage. Thus, the motivation behind this study is to make homemade phenol-based reagent for affordable research.

## Materials and Methods

### Preparation of Phenol-Based Reagent

#### Saturated Phenol

Phenol has a limited solubility in water (8.3 g/100 ml). It is slightly acidic: the phenol molecule has weak tendencies to lose the H^+^ ion from the hydroxyl group. The pH of water saturated phenol solution is likely to be around 5–6 which is not optimal for getting pure RNA without DNA contaminants. Therefore, it is necessary to prepare acidic phenol solution of pH around 3–4 as an initial step of composing phenol-based reagent. Phenol buffer solution was adjusted to the pH of solution at pH 4.2. In the present work, citrate buffer solution was used as acidic buffer. Then, acidic phenol was prepared by mixing equimolar amount of citric acid and its conjugate base sodium citrate as the following procedure:

i. The pure phenol crystal was melted in water bath at 65 ^∘^C.
ii. An equal volume of 0.5 M citrate buffer solution was added.
iii. At ambient temperature, the solution was mixed for 15 min.
iv. The phases were allowed to be separated at 65 ^∘^C and siphoned off the top layer (remaining non-mixed buffer) and discarded.
v. Previous steps (2–4) were repeated 3 times.

### Preparation of Phenol-Based RNA Extraction Reagent

Phenol-based reagent was prepared in the following formula under fume hood: 38% acidic phenol, 0.8 M guanidine thiocyanate, 0.4 M ammonium thiocyanate, 0.1 M sodium acetate, and 5% glycerol. The preparation method was recruited from different references (Chomczynski 1993; Chomczynski and Sacchi 1987; Dooley and Castellino 1972; Rio et al. 2010).

### Biological Samples

#### Cell Culture

Hepatocellular carcinoma HuH-7 cell line was obtained from a distributing company for biological products & vaccines (VACSERA), Cairo, Egypt. Cells were passaged in humidified tissue culture incubator at 37 ^∘^C and 5% CO_2._ HuH-7 was maintained in High glucose-DMEM with 10% fetal bovine serum, l-glutamine, and 1% penicillin/streptomycin.

#### Fungal Samples

*Aspergillus niger* (isolated from soil) was cultured on SDA for 3 days at 28 °C. Fungal growth scarped from 0.5 cm agar plugs was transferred to 0.5 ml phenol-based reagent. Mechanical disruption of cell walls using sterile toothpicks for 1 min was performed on part of samples. Some samples were not processed with mechanical disruption as a comparison. RNA extraction was tested using both mycelium and conidia, the aim was not to obtain exclusively one form.

#### Bufo regularis

*Bufo regularis* is a common toadspecies in Egypt. Therefore, it is widely used to teach anatomy for undergraduate students. Kidney, pancreas, heart, liver, testis, and stomach were harvested from an already dissected toad used in anatomy laboratory to reduce the use of animals. Tissues were subjected to homogenization using scalpel and syringe in phenol-based reagent.

#### Tumor Samples

Forty breast cancer and the adjacent normal tissue samples were obtained from the National Cancer Institute with written informed consent from each patient or their legal guardian. The study was conducted in compliance with the ethical guidelines established by the National Cancer Institute Cairo University, which included thorough review of the study's design and objectives, as well as the protection of patient rights and confidentiality. The study was approved by the Committee, and all procedures were carried out in accordance with the approved protocol.

A written informed consent was signed from each patient or their legal guardians. Our study was approved by the ethical committee of the National Cancer Institute Cairo University (Ethical approval number 201516025.2).

### RNA Purification Using the Homemade Phenol-Based Reagent

**Fig. 1:**
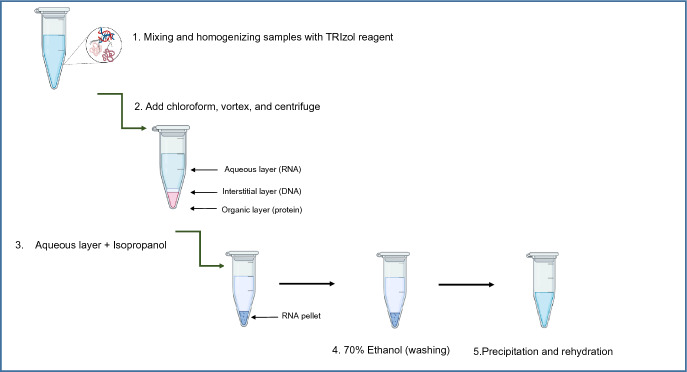
Schematic overview of RNA purification using phenol-based reagent

Samples were firstly homogenized either by syringe needles for tissues, cell lines were mixed with Triphasic phenol, and mixing with/without mechanical pressure for fungi. At the end of this step, investigated samples were mixed/homogenized in 0.5ml of Triphasic phenol reagent. To the cell lysate, 100µl of chloroform was added and mixed vigorously by vortex at room temperature. The mixture was then incubated for 2–3 min at room temperature. In order to separate the RNA from DNA and proteins, cell lysate was centrifuged at 13,000×*g* for 10 min at ambient temperature. The upper aqueous layer was transferred to new tube and mixed with 250 µl isopropanol. To enhance the RNA precipitation, isopropanol-lysate mixture was kept at – 20 °C for 30 min, followed by maximum speed centrifugation at room temperature. The isopropanol was discarded and the RNA pellet was washed with 70% ethanol. The washed pellets were finally dissolved in DEPC-treated water and incubated 10 min at 70°C. A scheme of the procedure is illustrated in Fig. 1.

The RNA purification was done in three biological repetitions. Concentration was measured using Nanodrop spectrophotometer (Thermoscientific). Average concentration was calculated for all experiments as well as the standard deviation. All experiments were done in comparison to commercial TRIzol (Thermoscientific). To check the quality of RNA, 1.5% agarose gel electrophoresis was performed to visualize 28s and 18s rRNA.

### cDNA Synthesis and RT-PCR

0.5 µg of RNA was reverse-transcribed to cDNA using GScript RTase (Genedirex). RT-PCR was performed for *GAPDH* gene expression. GAPDH forward and reverse primers were used for amplification as the following: CTGGGCTACACTGAGCACC and AAGTGGTCGTTGAGGGCAATG. The amplicon was visualized by electrophoresis on 1.5% agarose gel.

## Results

### Homemade Phenol-Based Reagent is Comparable to the Commercial One

**Fig. 2:**
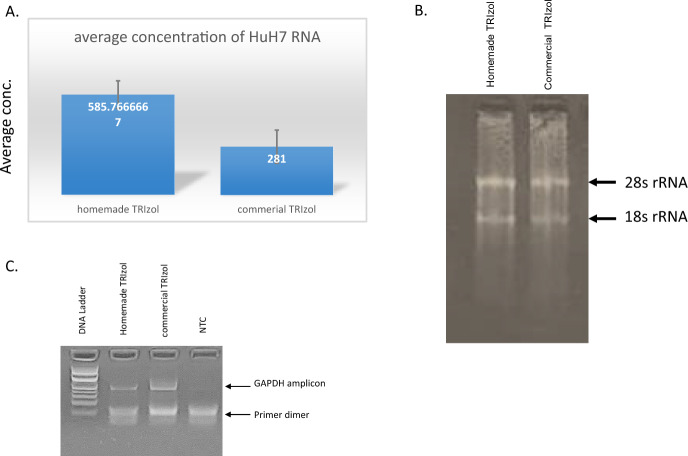
Total RNA purification from HuH7 cell line. **A** The histogram represents the average concentrations of purified RNA using homemade and commercial TRIzol reagent. Error bars represents standard deviation of experimental repeats. **B** Total RNA integrity was tested by 1.5% agarose gel electrophoresis to detect 28s and 18s rRNA. **C** RT-PCR for GAPDH was performed using cDNA from RNA templates of homemade and commercial TRIzol

Total RNA purification was executed from different biological samples as described in the materials and methods section. At least three repetitions were performed for each biological sample. In addition, each experiment was done in parallel to Trizol from Thermoscientific. The concentration of RNA samples were measured using Nanodrop spectrophotometer to compare the final yield of total RNA. The average concentration was compared for all repetitions. Accordingly, in HuH-7 cell line the concentration of purified RNA using our homemade phenol-based reagentis higher than the commercial TRIzol. In fungal samples, the yield was slightly higher using commercial TRIzol as shown in Fig. 2. Also, GAPDH mRNA was successfully amplified using RT-PCR (Fig. 2C).

### Fungal Samples Need Mechanical Disruption Before RNA Purification

**Fig. 3:**
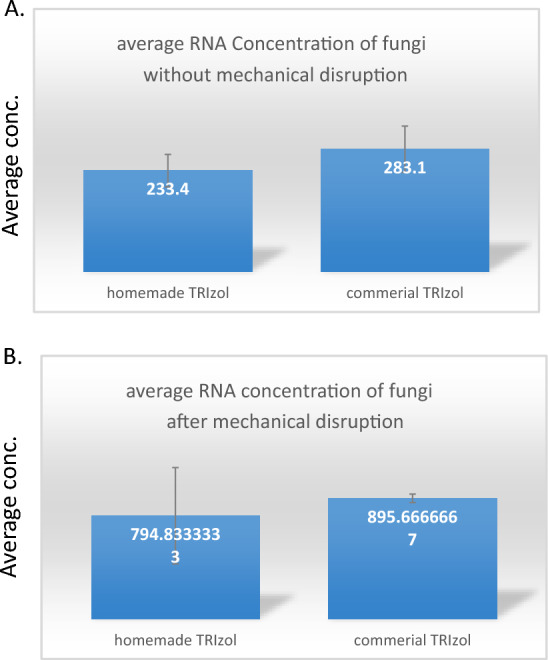
Average concentration of purified RNA from *Aspergillus niger.* **A** Without mechanical corruption of the fungal sample. **B** With mechanical corruption

**Fig. 4:**
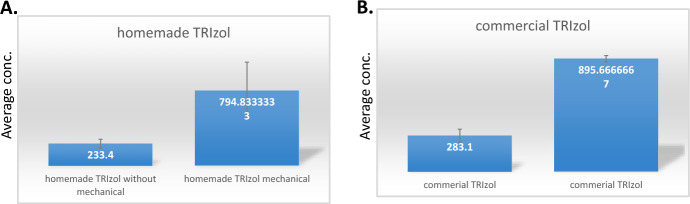
Effect of mechanical corruption of fungi on the yield of total RNA. **A** Homemade phenol-based reagent. **B** Commercial TRIzol

Due to the presence of cell wall in fungi, a mechanical disruption was needed before total RNA purification. Since the mechanical disruption is time consuming, we tried the purification without mechanical disruption as we did believe in the strength of Guanidine thiocyanate to denature cell walls. The RNA purification was successful in both cases (with/without mechanical disruption) as shown in Fig. 3. However, the mechanical  disruption  did enhance the purified RNA concentrations by 3.4- and 3.2-fold using both homemade and commercial TRIzol, respectively, as shown in Fig. 4.

### Total RNA is Successfully Purified from Tissues Using Homemade Phenol-Based Reagent

**Fig. 5:**
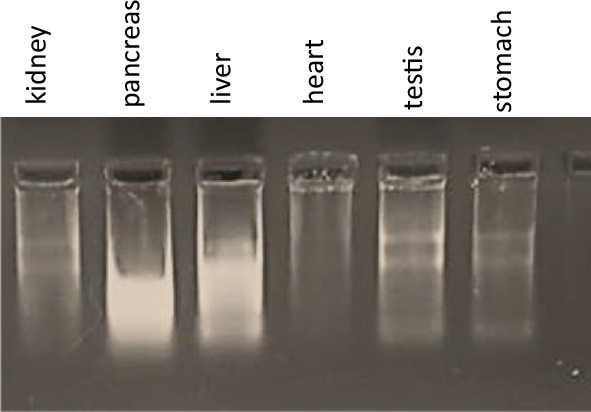
Purified total RNA from *Bufo regularis* (different organs) using homemade phenol-based reagent

RNA was successfully purified from kidney, pancreas, heart, liver, testis, and stomach harvested from the toad *Bufo regularis* (Fig. 5).

**Fig. 6:**
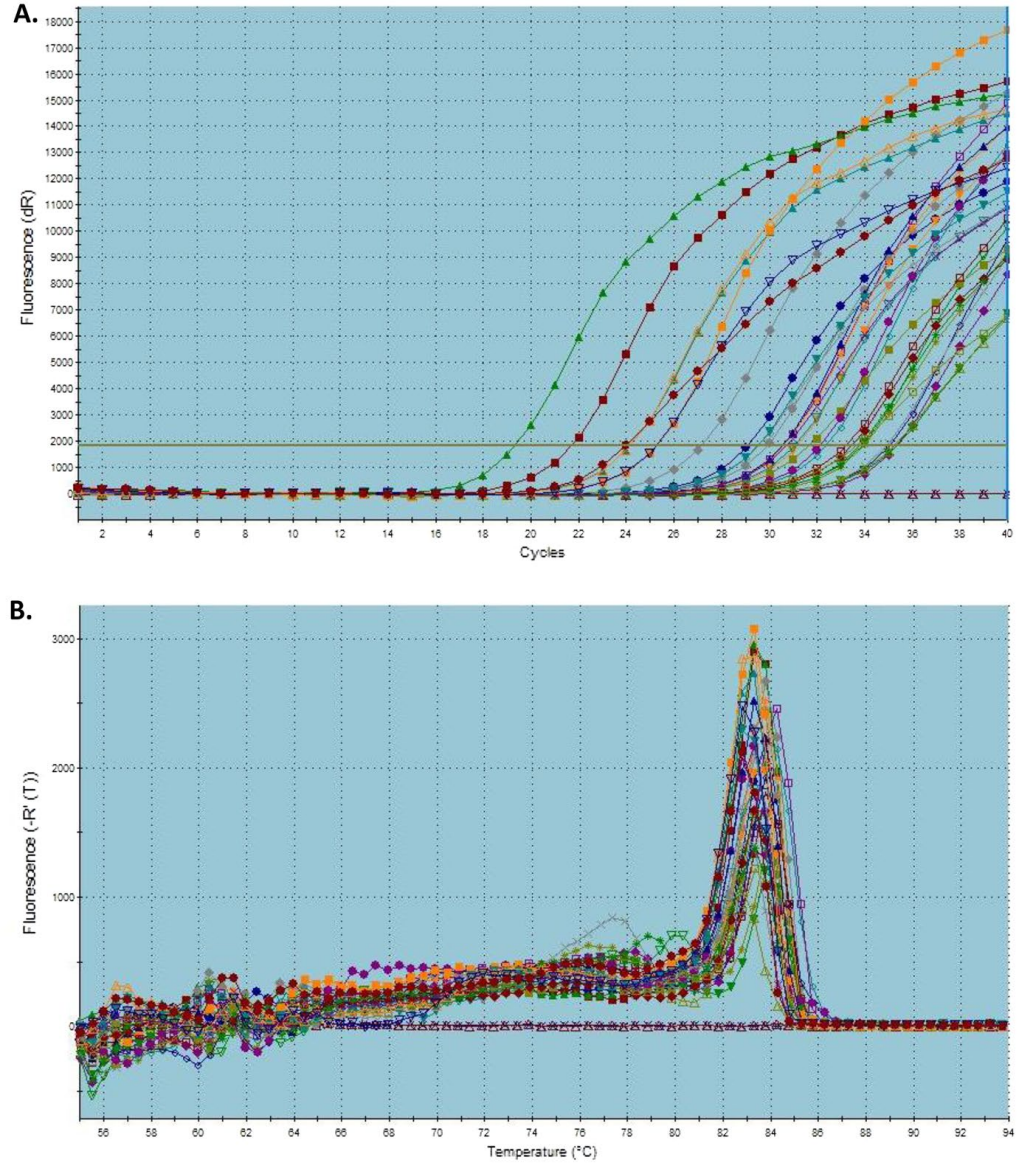
qRT-PCR results of 40 breast cancer patients. **A** Amplification curves, **B** dissociation curves

In addition, eighty tissue samples were collected from forty breast cancer patients; tumor sample and its adjacent tissue were excised from each patient. The RNA was purified competently using the homemade reagents. The concentration and purity was executed using nandrop spectrophotometer as shown in Table 1. Also, qRT-PCR for GAPDH gene was performed; amplification was effectively obtained with their dissociation curves (Fig. 6).

**Table 1:** The results of 80 samples obtained from forty breast cancer patients

	Excised tissue	Concentration (ng/μl)	OD260/280 ratio
1	N	702.2	1.78
	T	251.4	1.61
2	N	410.7	1.56
	T	786.1	1.8
3	N	877.9	1.84
	T	426.5	1.66
4	N	541.3	2
	T	208.1	1.56
5	N	659.8	1.81
	T	378.2	1.75
6	N	305.9	1.89
	T	263.6	1.63
7	N	216.4	1.43
	T	366.1	1.55
8	N	1317.6	1.49
	T	283.9	1.5
9	N	735.8	1.68
	T	396.5	2.1
10	N	464.2	1.85
	T	908	1.52
11	N	404.6	1.55
	T	920.3	1.6
12	N	692.1	1.69
	T	376.4	1.77
13	N	582	1.95
	T	1062.1	2.01
14	N	228.6	1.44
	T	1014.7	1.83
15	N	773.3	1.54
	T	923.5	1.81
16	N	441.8	1.37
	T	639	1.6
17	N	771.7	1.94
	T	325.1	1.4
18	N	1008.3	1.81
	T	1807.9	1.72
19	N	438.2	1.54
	T	718.4	1.68
20	N	1590.5	1.56
	T	1910.2	1.5
21	N	589.9	1.74
	T	422.7	1.9
22	N	309.6	1.55
	T	967	1.65
23	N	803.8	1.81
	T	347.6	1.41
24	N	634.2	1.38
	T	1178.1	1.84
25	N	313.5	1.6
	T	1406.7	1.78
26	N	391.9	1.55
	T	828.2	1.71
27	N	448	1.52
	T	479.5	1.74
28	N	563.2	1.85
	T	329.4	1.69
29	N	1758.1	2.12
	T	399.7	1.57
30	N	581.1	1.69
	T	256.8	1.36
31	N	1837.9	1.64
	T	488.4	1.72
32	N	960.7	2.08
	T	339.4	1.86
33	N	369.5	1.6
	T	246.8	1.47
34	N	611.2	1.93
	T	724.5	1.72
35	N	1407.3	1.81
	T	394.5	1.58
36	N	868.7	2.1
	T	598.1	1.52
37	N	887.9	1.49
	T	537.4	1.58
38	N	284.1	1.39
	T	335.2	1.47
39	N	490.6	1.66
	T	536.9	1.84
40	N	985.7	1.92
	T	819.4	1.53

RNA concentrations and purity are presented as obtained from the Nanodrop spectrophotometer

## Discussion

Since year 2020, there has been  a big dilemma in molecular biology consumables market. There is an international stream to allocate academic research budgets to Covid-19 pandemic which makes sense in the current international crisis. The other side of the current situation is the delays in shipping of laboratories’ purchases. In the last two years, several research projects were carried out in our lab to make homemade solutions, in order to compromise with need gap (Abdelfattah et al. 2022; Elnagar et al. 2022; Mahmoud et al. 2022). Hence, the aim of the present work is to make feasible homemade phenol-based reagents for RNA purification.

Phenol-based reagent is guanidine thiocyanate mixed with acid phenol. Guanidine thiocyanate is a chaotropic agent which strongly denature proteins including RNase. It is an advantageous solution, since it is a single-step RNA purification from different biological sources. It separates  RNA, DNA, and proteins in triphasic state after adding chloroform. Therefore, phenol-based reagent is widely used in molecular biology laboratories for different purposes (Chomczynski 1993; Chomczynski and Sacchi 1987; Dooley and Castellino 1972; Hummon et al. 2007; Rio et al. 2010; Tan and Yiap 2009). Phenol-based reagent  is widely employed to purify RNA from plants, mammalian cell lines, tumor tissues, and was also useful in Covid-19 pandemic (Connolly et al. 2006; El Leithy et al. 2015; Li et al. 2009; Abdelfattah et al. 2021; Meng and Feldman 2010; Miranda-Ortiz et al. 2021; Villota et al. 2021).

For a minimum viable extraction solution, a homemade phenol-based reagent was composed using homemade acidic phenol, guanidine thiocyanate, and other components. For proof of concept, the homemade phenol-based reagent was compared to the commercial one to purify total RNA from different sources of biological samples. Our results showed successful RNA purification. In HuH7 cell line, using same number of cells, RNA yield in nanograms was higher using homemade phenol-based reagent. Also, the mRNA was successfully amplified using RT-PCR which reflects the quality of RNA. For further testing of homemade phenol-based reagent, RNA was purified from tissue samples from toad.

Fungi have compact cell walls consisting of (1–3)-b d-glucan, (1,6)-b-glucans, chitin, lipids, peptides and sometimes melanin that obstruct lysis and the recovery of nucleic acids due to their resistance to enzymatic digestion and chemical breakdown, thus a separate step of mechanical disruption of cell walls is usually required (Fredricks et al. 2005; Rodrigues et al. 2018). Thus, mechanical disruption was used in our present work and compared to the lysis of cell walls in phenol-based reagents only without mechanical disruption. Consistently with the previously mentioned studies, the RNA concentrations is increasing by at least 3 times after mechanical disruption.

In order to use our homemade phenol-based reagent, RNA purification was executed using eighty samples representing 40 breast patients (each patient with biopsies of tumor sample and its adjacent normal tissue). Concentrations and 260/280 ratio were measured, which were the primary indicator of RNA quantity and purity. For further validation, *GAPDH* gene was selected as a housekeeping gene with basal expression in all kind of tissues. So, the good RNA integrity is indicated with early CT value of the housekeeping genes.

## Conclusion

In conclusion, homemade phenol-based reagent is comparable to the commercially available reagents, as it was successful to purify RNA from cell line, different tissues, and fungi. Also, from the cost perspective, homemade solutions is about one-tenth of the commercial TRIzol cost. Hence, it is more affordable for researchers to prepare their own phenol-based reagents, especially those in developing countries.

## Data Availability

The datasets used and/or analyzed during the current study are available from the corresponding author on reasonable request.
